# Serotonin stimulates proliferation of ionocytes via the 5-HT2A receptor in zebrafish larvae

**DOI:** 10.1242/jeb.251808

**Published:** 2026-06-15

**Authors:** Willamina M. MacDonald, Ethan P. Gillespie, Michael G. Jonz

**Affiliations:** Department of Biology, University of Ottawa, 30 Marie Curie Pvt., Ottawa, ON, Canada, K1N 6N5

**Keywords:** Serotonin, 5-HT2A receptor, Ionocyte, Zebrafish, Proliferation, Environmental acidification

## Abstract

Osmoregulation is an essential process in all living organisms. For aquatic organisms, such as freshwater fishes whose natural environment is hypoosmotic, specialized cells, called ionocytes, are present in the skin during developmental stages and contribute to the maintenance of osmotic homeostasis. Such cells are known to proliferate in response to osmotic or ionic stress, but the molecular mechanism by which that process is regulated remains poorly characterized. In this study, using immunohistochemistry and confocal microscopy, we demonstrate that cutaneous ionocytes in developing zebrafish (*Danio rerio*) express serotonin 2A (5-HT2A) receptors by co-labelling with other known ionocyte markers, such as the Na^+^/K^+^-ATPase, Concanavalin A and Mitotracker. Furthermore, by quantifying ionocyte number through early stages of development, we implicate 5-HT2A receptors in initiating ionocyte proliferation. Exposure of zebrafish embryos and larvae to acidic pH, or exogenous 5-HT, increased the number of cutaneous ionocytes. The effects of both stimuli were abolished in the presence of the 5-HT2A receptor-specific antagonist, ketanserin. Moreover, activation of 5-HT2A receptors led to increased detection of ionocytes with phosphorylated extracellular signal-regulated kinase (ERK), a key regulator of cell division and differentiation linked with 5-HT2A. We used tetrabenazine, an inhibitor of vesicular monoamine transporter 2 (vmat2) and 5-HT storage, to deplete potential sources of 5-HT. Tetrabenazine treatment in fish exposed to acidic pH reduced ionocyte proliferation, implicating a source of 5-HT that uses vmat2 in the regulation of ionocyte populations. These results demonstrate the importance of a pathway initiated by 5-HT2A activation that regulates ionocyte proliferation in developing zebrafish exposed to environmental acidification.

## Teaser

**Summary:** Serotonin stimulates 5-HT2A receptors on ionocytes, leading to proliferation upon acclimation to acidic environments in zebrafish larvae.

## INTRODUCTION

All living organisms must be equipped with mechanisms to maintain osmotic homeostasis. In the case of freshwater fishes, whose natural environment is hypoosmotic, osmoregulation includes excretion of dilute urine following reabsorption of ions in the kidney, and active absorption of ions through specialized cells called ionocytes (Evans et al., 2005). Ionocytes can be classified based on the ion transporters they express, many of which are analogous to transporters in renal tubular cells, the principal site of osmotic regulation in terrestrial vertebrates (Hwang and Chou, 2013). In zebrafish (*Danio rerio*), ionocytes are present in the gills of adults and the skin of developing fish. In both the gills and the skin, at least five populations of ionocyte have been identified: cells that are H^+^-ATPase rich (HR) or Na^+^/K^+^-ATPase rich (NaR), those that express the Na^+^/Cl^−^ cotransporter (NCC) or solute carrier 26 (SLC26), and K^+^-secreting (KS) cells (Guh et al., 2015). Ionocytes in the skin of developing zebrafish are especially convenient to study as they are directly exposed to the environment and are easily observed *in situ* with whole-mount preparations (Hwang and Chou, 2013).

In response to osmotic stress, ionocytes increase in number and in their expression of ion transporters. Cutaneous HR ionocytes of zebrafish larvae have previously been shown to proliferate with exposure to acidic (Horng et al., 2009; Shir-Mohammadi and Perry, 2020) and low Na^+^ environments (Shir-Mohammadi and Perry, 2020). It has also been demonstrated that individual HR ionocytes increase their H^+^-secreting function in response to acidic environments (Horng et al., 2009). The molecular mechanisms underlying these phenomena have yet to be fully characterized. Thus far, hormones such as prolactin (Breves et al., 2014) and isotocin (Chou et al., 2011) have been proposed as major regulators of ionocyte number. Furthermore, cortisol has been demonstrated to play a role in the proliferation of gill ionocytes and an increase in Na^+^/K^+^-ATPase activity, at least partially by direct stimulation (Evans et al., 2005). With respect to the increase in function of single HR cells, adrenergic systems have been suggested to play a role. It has been demonstrated that HR cells express β-adrenergic receptors, and that Na^+^ uptake in these cells is altered by acute treatment with adrenergic agonists and antagonists (Kumai et al., 2012). These findings clearly demonstrate that regulation of ionocyte number and function depends on multiple factors that play a role in stress responses, raising the question of whether additional hormones or neurotransmitters that mediate stress responses in fishes are involved.

One such neurotransmitter is serotonin (5-hydroxytryptamine, 5-HT), which has been shown to become elevated in the brain of zebrafish exposed to hypoxic or osmotic stressors (Sreelekshmi et al., 2022). Increased 5-HT levels have also been observed in the brain (Boeck et al., 1996) and in the gills (Mustafayev and Mekhtiev, 2008) of other species of fish exposed to high salinity. An increase in 5-HT levels in the gill is especially interesting because ionocytes are located mainly in the gills of adult fishes, and are in close proximity to neuroepithelial cells (NECs) that retain and release 5-HT (Jonz and Nurse, 2006; Reed et al., 2025). Whether an increase in 5-HT levels occurs in the skin of zebrafish larvae exposed to osmotic stressors has not been demonstrated, though cutaneous, 5-HT-containing NECs are also present (Coccimiglio and Jonz, 2012) and are adjacent to cutaneous ionocytes (Dean et al., 2017). Given that acute treatment of zebrafish larvae with 5-HT does not lead to changes in Na^+^ uptake (Kumai et al., 2012), it is unlikely that 5-HT affects the expression or activity of ion transporters at the level of single ionocytes. However, it has yet to be investigated whether 5-HT might lead to the proliferation of ionocytes.

5-HT has previously been demonstrated to be involved in the regulation of cell number, with effects on cell proliferation, maturation and apoptosis in various cell types and organisms including cells of the lung, kidney and nervous system (Azmitia, 2001). Interestingly, it has been demonstrated in rats that 5-HT induces the proliferation of renal mesangial cells (Takuwa et al., 1989) mediated by 5-HT2A receptors through pathways involving extracellular signal-regulated kinases (ERKs) (Göőz et al., 2006; Greene et al., 2000; Grewal et al., 1999).

The present study employed immunohistochemistry and confocal microscopy to reveal that serotonin 2A (5-HT2A) receptors are found in cutaneous ionocytes in zebrafish larvae. We hypothesized that 5-HT2A receptors facilitate the regulation of ionocyte number in response to environmental acidification. To test this hypothesis, we first aimed to demonstrate that 5-HT2A immunolabelling co-localized with known markers of multiple ionocyte subtypes. We then aimed to demonstrate a role for 5-HT, via the 5-HT2A receptor, in regulating ionocyte number. To do so, we quantified the number of ionocytes under different conditions, including acid acclimation and treatment with 5-HT2A-specific agonists and antagonists. Furthermore, through pharmacological inhibition of vesicular monoamine transporter 2 (vmat2), we demonstrate that the source of 5-HT is a cell type that utilizes vmat2, potentially NECs, which we have shown become depleted of 5-HT upon inhibition of vmat2. Finally, using an antibody raised against phosphorylated ERK, we provide evidence that the pathway by which the 5-HT2A receptor leads to the proliferation of ionocytes involves ERK and that phosphorylated ERK is present only in 5-HT2A-positive ionocytes that have been stimulated by 5-HT.

The results highlight a role for 5-HT, via 5-HT2A receptors, in initiating proliferation of ionocytes in response to low pH environments. This novel role for 5-HT in zebrafish larvae contributes to our evolving understanding of the mechanisms by which zebrafish respond to environmental acidification at early developmental stages. These findings may have implications for understanding how fish respond to osmotic challenges in the environment and in aquaculture, as well as potential applications for zebrafish as a model organism to enhance our understanding of analogous cell types and pathways in mammals.

## MATERIALS AND METHODS

### Animal care

Zebrafish, *Danio rerio* (F. Hamilton 1822), were maintained at the Laboratory for the Physiology and Genetics of Aquatic Organisms, University of Ottawa, at 28°C on a 14 h:10 h light:dark cycle (Westerfield, 2007). Water entering the facility from the city of Ottawa was dechloraminated and aerated. System water pH was buffered using ProLine^®^ sodium bicarbonate (NaHCO_3_), following the manufacturer's directions (cat. no. PC12, Pentair Aquatic Eco-Systems, Inc., Apopka, FL, USA). Wild-type male and female zebrafish were used for all experiments labelling ionocytes. In experiments labelling NECs, an ET(vmat2:GFP) transgenic line was used. ET(vmat2:GFP) zebrafish express green fluorescent protein (GFP) under the vmat2 promoter (Wen et al., 2008), and have previously been demonstrated to label cutaneous NECs in larval zebrafish (Pan et al., 2021). Embryos were bred from 12 month adults using standard breeding techniques (Westerfield, 2007) and transferred to 150 mm Petri dishes containing buffered system water and 3.0×10^−5^% Methylene Blue (unless otherwise stated) at 0–2 h post-fertilization (hpf). Dishes were kept in an incubator at 28.5°C and media was replaced daily. When the desired age (2–7 days post-fertilization, dpf) was reached, larvae were euthanized in 1 mg ml^−1^ MS-222 buffered with 0.5 mg ml^−1^ NaHCO_3_ at 4°C. All animal use procedures were carried out according to institutional guidelines and protocol BL-3666 in accordance with the Canadian Council on Animal Care.

### Immunohistochemistry

Euthanized zebrafish larvae were processed for whole-mount immunohistochemistry according to previously established protocols (Coccimiglio and Jonz, 2012; Jonz and Nurse, 2005). In brief, samples were processed by: (1) fixation, (2) permeabilization, (3) incubation in primary antibodies and (4) incubation in secondary antibodies. Between each step, the larvae were rinsed 3 times for 3 min in phosphate-buffered solution (PBS) containing (mmol l^−1^): NaCl 137, Na_2_HPO_4_ 15.2, KCl 2.7 and KH_2_PO_4_ 1.5 at pH 7.8. Fixation was done in 4% paraformaldehyde (PFA) in PBS at 4°C overnight. Permeabilization was then accomplished by incubating the samples in 2% Triton X-100 (TX-100) in PBS at 4°C for 48 h. Primary and secondary antibodies were prepared by dilution in PBS to 1:100. Fish were incubated in primary antibodies at 4°C for 24 h. Samples were then incubated in secondary antibodies for 1 h at room temperature in a dark chamber. In double-labelling experiments, antibodies were used in combination. Finally, samples were mounted on glass slides in ProLong Diamond Antifade Mountant (cat. no. P36970, Thermo Fisher Scientific, Waltham, MA, USA).

### Antibodies and fluorescent probes

To demonstrate that 5-HT2A receptors were present in ionocytes, a 5-HT2A receptor antibody was used. The rabbit polyclonal 5-HT2A antibody (RRID_AB2807092, cat. no. PA5-95288, lot nos 79627209 and ZG4406664A, Thermo Fisher Scientific) was raised against a synthetic peptide corresponding to the C-terminus of human 5-HT2A receptor, specifically amino acids 400 to 431 (manufacturer specifications). Based on sequence alignment to the antigen sequence (Fig. S1), the antibody is expected to bind all 5-HT2A paralogues and isoforms in zebrafish. Therefore, with this antibody it is not possible to ascertain which specific 5-HT2A paralogue(s) or isoform(s) is expressed in ionocytes. Sequence alignments were also performed between the antigen sequence of the 5-HT2A antibody and other 5-HT2 (5-HT2B or 5-HT2C) receptors in zebrafish. No alignment between the antigen sequence and 5-HT2B or 5-HT2C receptors was observed, suggesting the 5-HT2A antibody is unlikely to bind to other 5-HT2 receptor subtypes.

To recognize the 5-HT2A antibody, two different secondary antibodies were used depending on the experiment: a goat polyclonal anti-rabbit Alexa 594-conjugated antibody (cat. no. A11012, lot nos 3112802 and 2616076, Thermo Fisher Scientific; magenta labelling) and a goat polyclonal anti-rabbit FITC-conjugated antibody (cat. no. 111-095-003, lot no. 104432, Jackson ImmunoResearch; green labelling). As the 5-HT2A antibody has not previously been used to label ionocytes in zebrafish larvae, we performed a negative control. To do so, we synthesized a custom peptide corresponding to the antigen sequence: KENKKPLQLILVNTIPALAYKSSQLQMGQKKN (lot no. ABc10124, ABclonal Science, Woburn, MA, USA). Primary antibodies were pre-incubated with the peptide at a concentration 5 times greater than that of the antibody at room temperature for 1 h before adding tissue. No immunolabelling was seen in pre-adsorbed samples, demonstrating that the antibody was not binding via off-target effects.

To provide evidence that the observed 5-HT2A-positive cells were ionocytes, they were co-labelled with well-established markers previously used to identify cutaneous ionocytes in zebrafish larvae. Such markers include an antibody raised against the α5 subunit of Na^+^/K^+^-ATPase, a known marker of NaR ionocytes (Hwang and Chou, 2013; Jonz and Nurse, 2006), Concanavalin A (ConA), a known marker of HR ionocytes (Esaki et al., 2007; Horng et al., 2009; Kumai et al., 2012; Kwong and Perry, 2016), and Mitotracker, a known marker of mitochondrion-rich cells (MRCs) including many ionocyte subtypes, namely NaR, HR and NCC ionocytes (Esaki et al., 2007; Kumai et al., 2012; Kwong and Perry, 2016). The mouse monoclonal α5 primary antibody (RRID: AB_2166869, Developmental Studies Hybridoma Bank, University of Iowa, Iowa City, IA, USA) was raised against Na^+^/K^+^-ATPase from chicken kidney and recognizes a cytosolic epitope on the α subunit of Na^+^/K^+^-ATPase (manufacturer specifications). It was recognized by a goat polyclonal Alexa 488-conjugated anti-mouse secondary antibody (cat. no. A11029, lot no. 57465A, Thermo Fisher Scientific).

ConA and Mitotracker are both chemicals conjugated to fluorophores to which live zebrafish larvae were exposed preceding immunohistochemistry. For experiments using Mitotracker to label ionocytes, Mitotracker Red CMXRos (cat. no. M7512; Invitrogen) was first dissolved in dimethyl sulfoxide (DMSO) (cat. no. D8418, Sigma) to a concentration of 1 mmol l^−1^ according to the manufacturer's directions. The stock solution was then diluted in extracellular solution (ECS) containing (mmol l^−1^): 120 NaCl, 5 KCl, 2.5 CaCl_2_, 2 MgCl_2_, 10 Hepes, 10 glucose at pH 7.8 to a final concentration of 300 nmol l^−1^ Mitotracker. Mitotracker exposures were performed in ECS as opposed to embryo medium to avoid potential adverse effects of Methylene Blue on mitochondria or uptake of Mitotracker dye. Methylene Blue has been shown to interact with mitochondria by multiple mechanisms (Klosowski et al., 2020). For experiments in which HR ionocytes were labelled with ConA, Concanavalin A-Alexa Fluor 488 conjugate (cat. no. C11252, Invitrogen) stock solutions were prepared to a final concentration of 5 mg ml^−1^ in 0.1 mol l^−1^ NaCO_3_ (pH 8.3) according to the manufacturer's instructions. The ConA stock was then diluted in ECS to a final concentration of 50 μg ml^−1^. Fish treated with Mitotracker or ConA were incubated in the desired dye for 30 min at 28.5°C before euthanasia and immunohistochemistry (described above).

To demonstrate activation of a pathway involving ERK, an antibody raised against phosphorylated ERK (p-ERK) was employed (mouse monoclonal anti-p-ERK, RRID: AB_2572926, cat. no. 14-9109-82, clone no. MILAN8R, lot no. 2643096, Invitrogen, Burlington, ON, Canada). Target specificity was previously validated in zebrafish larvae, where the p-ERK antibody was used as a biomarker for neuronal activation (Spadacini, 2024). In the present study, p-ERK labelling in the absence of other antibodies was done to test for cross-reactivity. Anti-pERK demonstrated the same labelling patterns with and without co-labelling with the 5-HT2A antibody. Anti-p-ERK was recognized with a goat polyclonal Alexa 594-conjugated anti-mouse secondary antibody (cat. no. A11005, lot no. 2043369, Invitrogen).

To quantify depletion of 5-HT from NECs following treatment with vmat2, an antibody raised against 5-HT was used (rabbit polyclonal 5-HT antibody, cat. no. S5545, lot no. 0000373540, Sigma-Aldrich, Oakville, ON, Canada). The anti-5-HT primary antibody was recognized with the goat polyclonal anti-rabbit Alexa 594-conjugated secondary antibody (cat. no. A11012, lot no. 3112802 and 2616076, Thermo Fisher Scientific). The 5-HT antibody has been extensively used to identify serotonergic NECs in the skin of zebrafish larvae (Coccimiglio and Jonz, 2012; Dean et al., 2017; Pan et al., 2021) as well as in zebrafish gills (Jonz and Nurse, 2003; Pan et al., 2021; Shakarchi et al., 2013). A mouse monoclonal antibody raised against GFP (RRID: AB_221568, cat no. A11120, lot no. 2052396, Thermo Fisher Scientific) was also used to enhance the endogenous GFP labelling in ET(vmat2:GFP) zebrafish to facilitate identification of NECs that express vmat2, as has previously been done (Pan et al., 2021). The anti-GFP primary antibody was recognized by a goat polyclonal anti-mouse Alexa 488-conjugated secondary antibody (cat. no. A11029, lot no. 57465A, Thermo Fisher Scientific).

### Acid acclimation

Developing zebrafish were exposed to low pH to induce ionocyte proliferation. For these experiments, embryos were transferred to acidic medium immediately after collection. Acidic medium was composed of system water supplemented with 300 μmol l^−1^ 2-(*N*-morpholino) ethanesulfonic acid hydrate (MES; cat. no. M8250, Sigma-Aldrich), adjusted to pH 4 with HCl, as done by Horng et al. (2009). Zebrafish were exposed immediately following embryo collection (0–2 hpf) for 2–7 days and kept in a 28.5°C incubator. Dishes contained no more than 60 embryos or larvae. Controls were treated in the same way but maintained in buffered system water.

Initial acid acclimation was done until developmental stages of 2–7 dpf to indicate which length of acid exposure would allow observation of robust increases in ionocyte number. Such experiments were done as one set of exposures with 5 fish sampled at each developmental stage and processed for immunohistochemistry. Following this initial experiment, all larvae were exposed to stimuli for 6 days (from 0 to 6 dpf) for experiments in which ionocyte number was quantified. In experiments determining 5-HT depletion in cutaneous NECs, zebrafish larvae were raised in acidic media for 4 days (from 0 to 4 dpf).

### Chemical exposure

To implicate the serotonergic system in ionocyte proliferation, zebrafish were exposed to 5-HT and the number of ionocytes on the trunk was quantified. To demonstrate that the effect of 5-HT was mediated by the 5-HT2A receptor, a 5-HT2A receptor-specific antagonist, ketanserin, was used. Finally, to begin to identify the source of 5-HT, a vmat2 inhibitor, tetrabenazine, was used. Tetrabenazine was used because NECs (a known source of 5-HT in the skin) use vmat2 to load 5-HT into vesicles preceding release upon stimulation. For experiments in which zebrafish larvae were treated with 5-HT (cat. no. B21263.03, Thermo Fisher Scientific), ketanserin (cat. no. S006, Sigma) or tetrabenazine (cat. no. T2952, Sigma), the appropriate medium (control or pH 4) was supplemented with 100 μmol l^−1^ of the drug of interest. The embryos were transferred to the appropriate medium immediately after collection (0–2 hpf) and exposed for 6 days (until 6 dpf). Solutions were prepared fresh daily when medium was changed. For solubility, ketanserin and tetrabenazine were first prepared as 100 mmol l^−1^ stock solutions in DMSO. The final concentration of DMSO in the medium was 0.1%, and controls were performed with 0.1% DMSO, where appropriate. For fish treated with both ketanserin and 5-HT, fish were pre-incubated with ketanserin for 1 h before the addition of 5-HT daily. There was no change in pH with the addition of 5-HT, ketanserin or tetrabenazine.

To confirm that tetrabenazine was depleting cutaneous NECs of 5-HT, the number of GFP-containing NECs in ET(vmat2:GFP) zebrafish that also stained positive for 5-HT was determined. This experiment was done at pH 7.0, and at pH 4.0 to confirm that the drug can deplete cutaneous NECs of 5-HT, and retains this effect at low pH. For these experiments, the chemical exposure protocol was done as described above, but a concentration of 50 μmol l^−1^ tetrabenazine was used at pH 7.0. Larvae were euthanized and processed for immunohistochemistry at 4 dpf as opposed to 6 dpf. This earlier developmental stage was chosen to facilitate quantification of cutaneous NECs as they are known to decrease in number as the gills develop.

### Imaging and analysis

For all experiments quantifying ionocytes, images were taken with an upright Olympus FV1000 BX61 LSM confocal microscope with a H101A ProScan platform equipped with continuous wave laser lines at 405, 488 and 559 nm. Images were viewed and captured with Fluoview software (Olypmus, Richmond Hill, ON, Canada). Images were taken in optical sections and rendered as stacks for rotation. For experiments quantifying 5-HT content of NECs, images were taken with a Zeiss Axio Observer A1 inverted microscope equipped with a QImaging QICAM Fast 1394 CCD camera (monochrome) and 3-colour continuous fluorescence: blue (365 nm, 420–470 nm), green (450–490 nm), red (534–558 nm, 575–640 nm). Images were captured with Northern Eclipse software (Empix Imaging, Mississauga, ON, Canada). Image processing was done in FIJI (Schindelin et al., 2012). Signals from red (Alexa 594 or Mitotracker) fluorophores were converted to magenta.

For experiments in which ionocyte number was quantified, images were taken at 40× magnification. Cells were identified manually and marked for quantification using the cell counter plugin in FIJI in three groups: cells labelled with Mitotracker only (magenta), cells labelled with 5-HT2A only (green) and co-labelled cells (white) for the quantification of ionocyte number. For quantification of p-ERK labelling, cells were counted in two groups: cells labelled with 5-HT2A only, and cells labelled with both 5-HT2A and p-ERK. Only cells on the trunk were counted. The fin fold contained very few ionocytes and was excluded from analysis. Only ionocytes were counted and were identified based on location and morphology. The only other 5-HT2A-labelled cells were neuromast cells, which were easily identified and excluded from further analysis.

For experiments in which NEC number was quantified, images were taken at 20× magnification. vmat2-positive NECs were identified based on GFP expression in the ET(vmat2:GFP) transgenic line and previously established morphological criteria (Coccimiglio and Jonz, 2012). To be classified as 5-HT positive, NECs had to clearly exceed background levels of fluorescence in the 5-HT (red) channel.

Statistical analysis was performed with Prism v.9.5.1 (GraphPad Software Inc., San Diego, CA, USA). For comparisons of cell number over the course of development and for the number of p-ERK-positive ionocytes or 5-HT-positive NECs, a Multiple Mann–Whitney test with the two-stage linear set-up procedure of Benjamini, Krieger and Yekutieli was used. For comparison of cell number at 6 dpf under different conditions, a Kruskal–Wallis test with Dunn's multiple comparison test was used. For comparison of the percentage of p-ERK-positive ionocytes or the percentage of 5-HT-positive NECs, a Mann–Whitney test was used. All data are presented as means±s.d. Sample size (*N*) refers to the number of fish and *P*<0.05 for all analyses.

## RESULTS

### 5-HT2A immunohistochemical labelling co-localizes with known markers of ionocytes

**Fig. 1.**
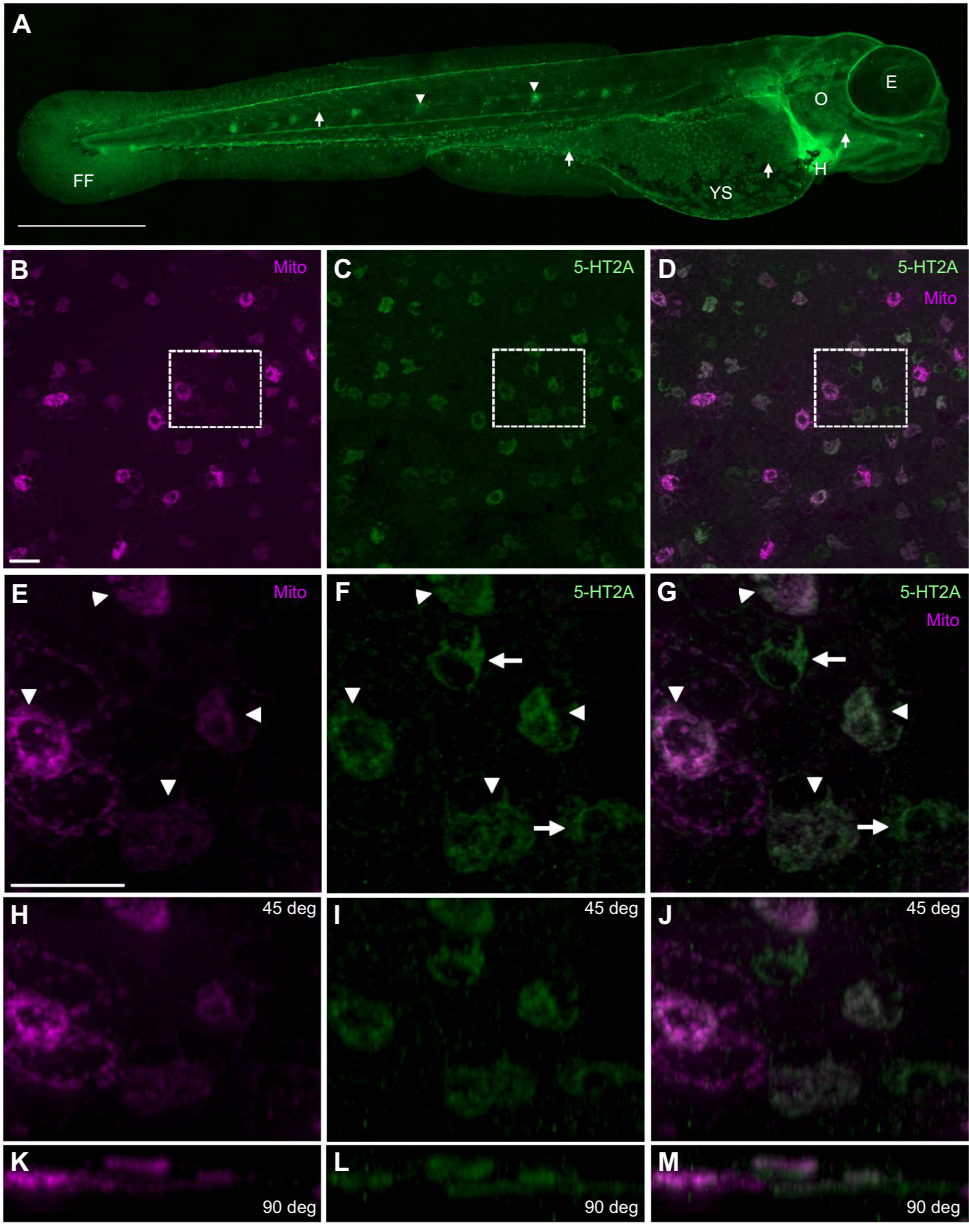
**Serotonin 2A receptor (5-HT2A) and Mitotracker labelling in zebrafish larvae.** (A) Representative confocal image of anti-5-HT2A labelling (green) in a zebrafish larva at 3 days post-fertilization (dpf). 5-HT2A immunohistochemical labelling consistently demonstrated this pattern in >180 fish between 2 and 7 dpf, alone and in combination with other markers. Images were taken at 10× magnification in three segments, which were aligned to create a montage of the entire fish. Scale bar: 500 μm. Arrows point to examples of cells characterized as ionocytes, and arrowheads to examples of neuromast cells. (B–M) Confocal images of Mitotracker (magenta) and 5-HT2A (green) labelling on the yolk sac of zebrafish larvae at 3 dpf. The 5-HT2A antibody and Mitotracker consistently demonstrated this pattern in 160 zebrafish between 2 and 7 dpf across multiple rounds of immunohistochemistry and different treatment conditions. B–D show an overview of Mitotracker and 5-HT2A labelling. E–G show an enlarged image of the boxed regions in B–D, with co-labelled cells (arrowheads) and cells positive for 5-HT2A only (arrows). H–J and K–M are images rotated back 45 and 90 deg. Scale bars in B (for B–D) and E (for E–M): 20 μm. YS, yolk sac; H, heart; E, eye; O, operculum; FF, fin fold; Mito, Mitotracker.

**Fig. 2.**
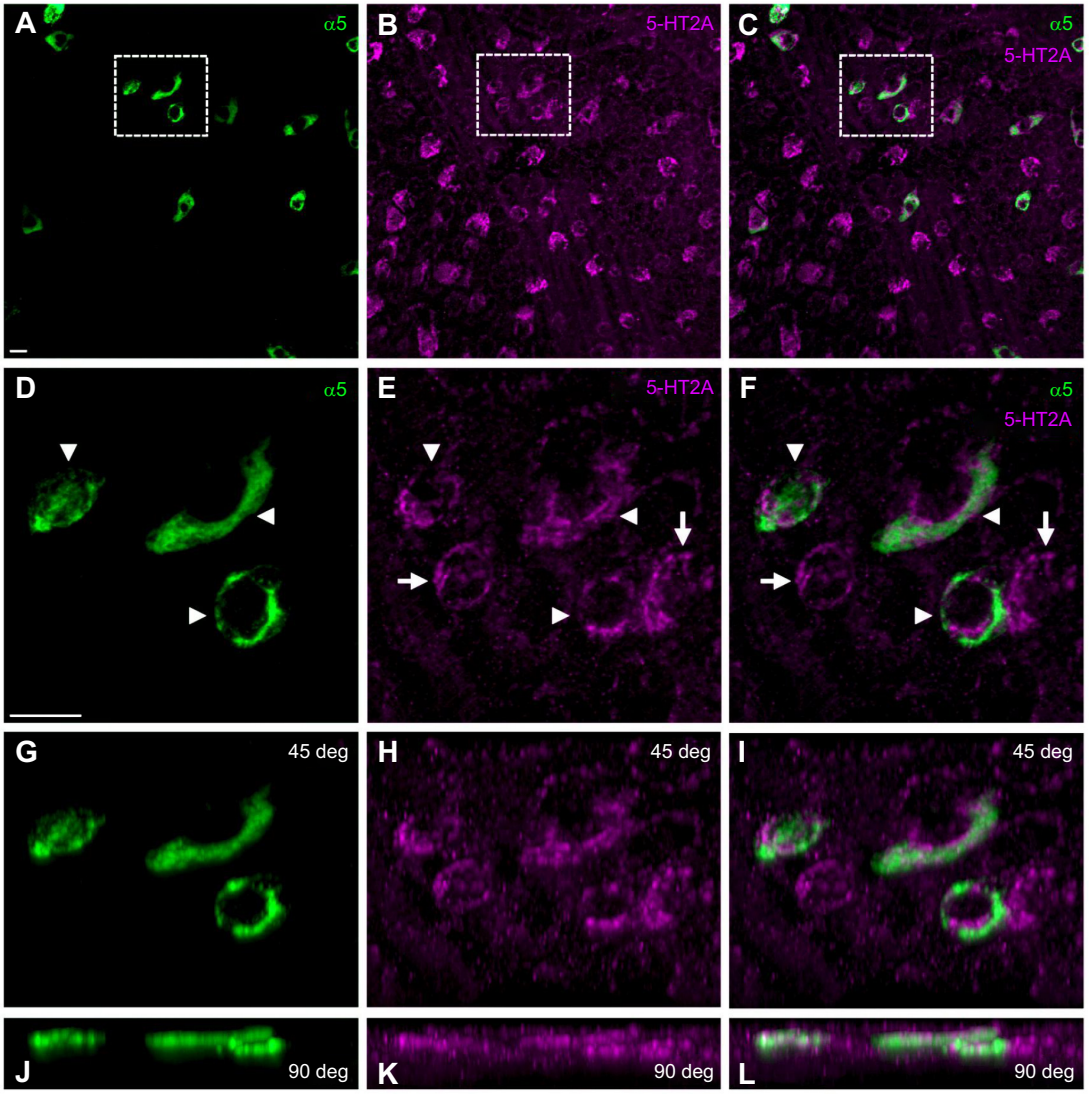
**Na^+^/K^+^-ATPase α-subunit (NaK α5)-positive ionocytes labelled positive for 5-HT2A.** Representative confocal images of α5 (green) and 5-HT2A (magenta) labelling in the yolk sac of zebrafish at 3 dpf. 5-HT2A and α5 antibodies consistently demonstrated this pattern in 25 zebrafish at 3 dpf across four rounds of immunohistochemistry. (A–C) Overview of α5 and 5-HT2A labelling. (D–L) Enlarged images of the boxed regions in A–C, showing co-labelled cells (arrowheads) and cells positive for 5-HT2A only (arrows). In G–I and J–L, images were rotated back 45 and 90 deg, respectively. Scale bars in A (for A–C) and D (for D–L): 10 μm.

**Fig. 3.**
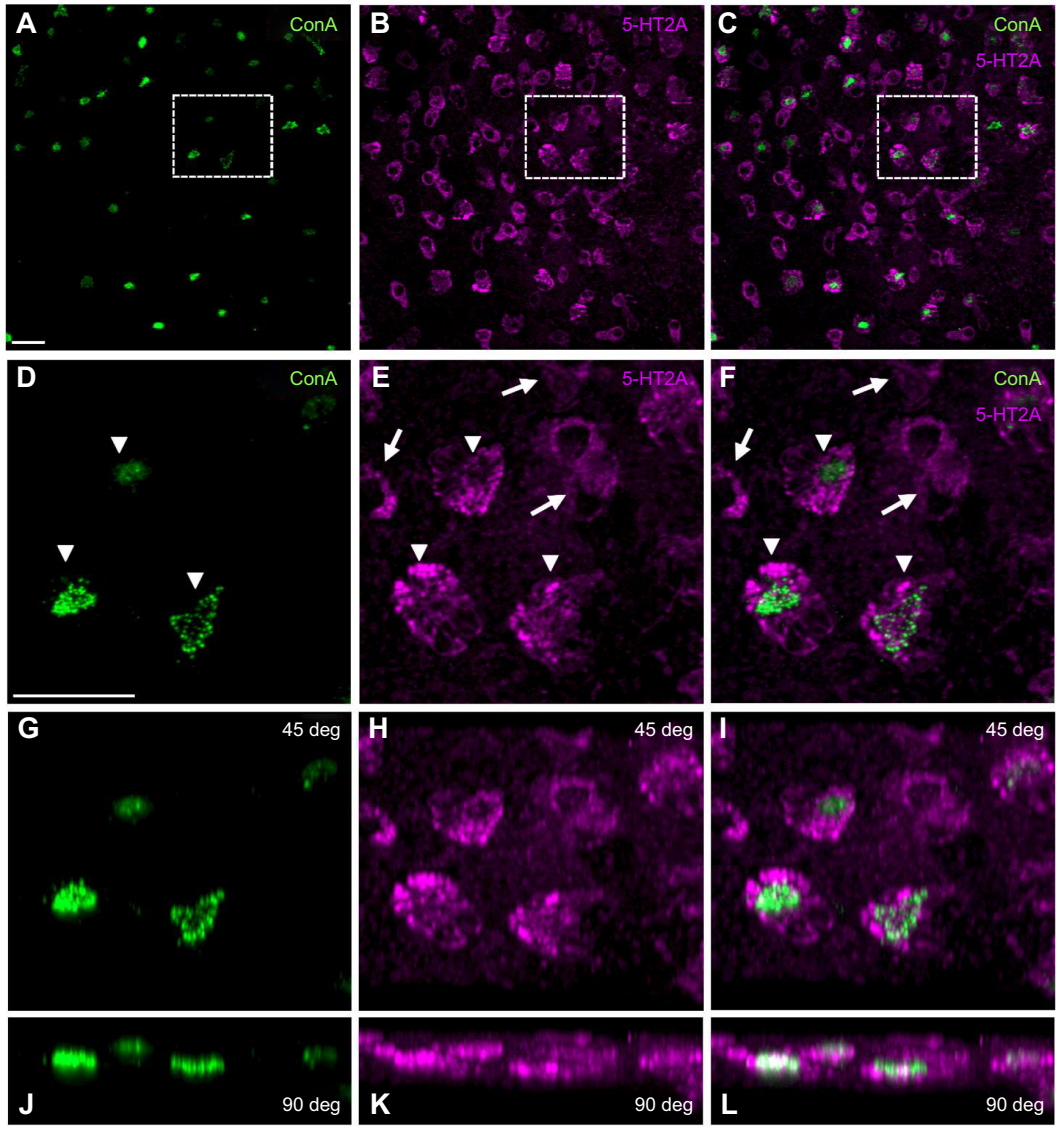
**H^+^-ATPase-rich (HR) (Concanavalin A-positive) ionocytes were positive for 5-HT2A.** Representative confocal images of Concanavalin A (ConA, green) and 5-HT2A (magenta) labelling on the yolk sac of zebrafish at 3 dpf. The 5-HT2A antibody and ConA consistently demonstrated this pattern in 15 zebrafish at 3 dpf across two rounds of immunohistochemistry. (A–C) Overview of ConA and 5-HT2A labelling. (D–L) Enlarged image of the boxed region in A–C, showing co-labelled cells (arrowheads) and cells positive for 5-HT2A only (arrows). In G–I and J–L, images were rotated back 45 and 90 deg, respectively. Scale bars in A (for A–C) and D (for D–L): 20 μm.

**Fig. 4.**
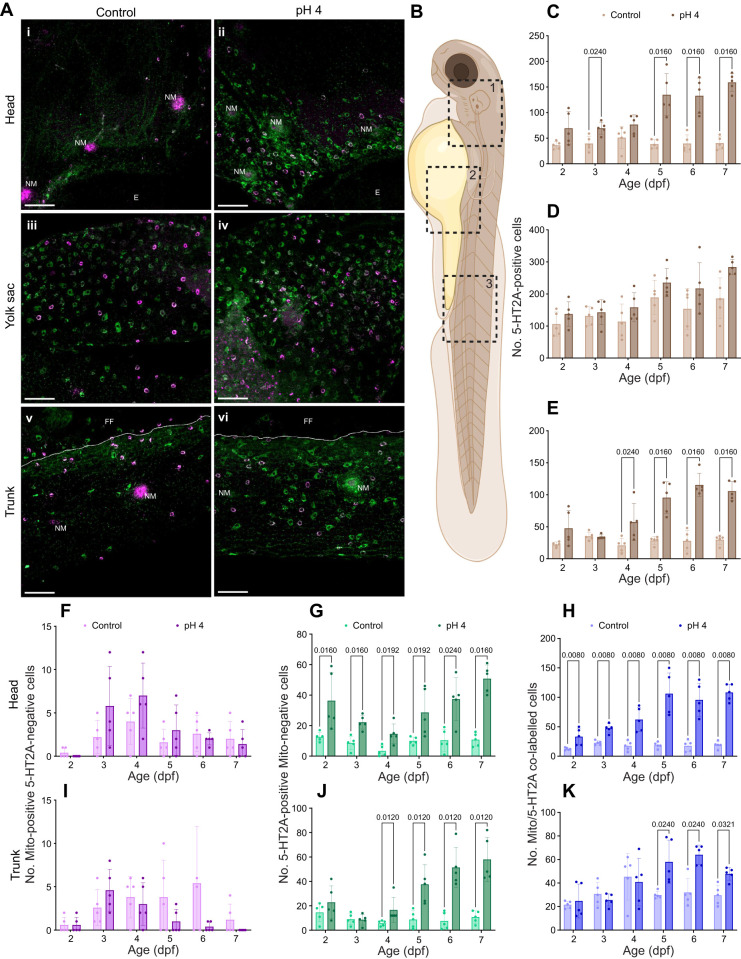
**The number of 5-HT2A-positive cells on the head and trunk increased with acid exposure.** (A) Representative confocal images of Mitotracker (magenta) and 5-HT2A (green) labelling on the head (i,ii), yolk sac (iii,iv) and trunk (v,vi) of zebrafish larvae at 7 dpf raised in control medium (left) or under pH 4 conditions (right). Scale bars: 50 μm. Five fish from each treatment group were processed daily from 2 to 7 dpf. Lines in v and vi depict the boundary set for quantification of ionocyte number on the trunk, excluding the fin fold. (B) Area imaged for the head (1), yolk sac (2) and trunk (3). Created in BioRender by Jonz, M., 2026. https://BioRender.com/066xsse. This figure was sublicensed under CC-BY 4.0 terms. (C–E) The total number of ionocytes with acid treatment compared with the control on the head (C), yolk sac (D) and trunk (E). (F–H) Cells labelled with Mitotracker only (F), 5-HT2A only (G) and co-labelled with 5-HT2A and Mitotracker (H) on the head. (I–K) Cells labelled with Mitotracker only (I), 5-HT2A only (J) and co-labelled with 5-HT2A and Mitotracker (K) on the trunk. Data were analysed using a Multiple Mann–Whitney test (two-tailed) with the two-stage linear set-up procedure of Benjamini, Krieger and Yekutieli. *P*-values are shown on the graph for groups that are significantly different (*P*<0.05, *N*=5 for each group at a given age). Data are means±s.d. NM, neuromast; E, eye; FF, dorsolateral fin fold.

Preliminary observation of 5-HT2A labelling in the skin of zebrafish larvae demonstrated ionocyte-like labelling patterns. At 3 dpf, the observed 5-HT2A-positive cells were distributed on the yolk sac and along the trunk, and began to appear on the head (Fig. 1). Therefore, we sought to co-localize the 5-HT2A antibody with known markers of ionocytes. The 5-HT2A antibody localized to the same cells as α5, ConA and Mitotracker. 5-HT2A and α5 labelling was generally confined to the plasma membrane, whereas Mitotracker was found in the cytoplasm, and ConA was localized to apical pits of HR cells, as previously described (Horng et al., 2009). Of the cells labelled with Mitotracker, 97% (*N*=160) also contained 5-HT2A (Fig. 1). Similarly, all observed cells labelled with α5 (Fig. 2) or ConA (Fig. 3) were also labelled with 5-HT2A (*N*=25 and *N*=15, respectively). Mitotracker, the most broad-spectrum marker of ionocytes employed, labelled the majority, approximately 76% (*N*=160), of the observed 5-HT2A-positive cells. The remaining 24% of 5-HT2A-positive cells not labelled with Mitotracker were ionocyte like, based on their morphology (Fig. 1), distribution (Fig. 1) and increase in number following acid exposure (Fig. 4). In subsequent experiments, we continued to quantify 5-HT2A-positive ionocyte-like cells with and without Mitotracker co-labelling separately.

### 5-HT2A-positive ionocytes increase in number with exposure of zebrafish larvae to acidic medium

To elicit ionocyte proliferation, we treated zebrafish larvae with acidic medium (Fig. 4). A significant increase (*P*<0.05, *N*=5) in the total number of 5-HT2A-positive ionocytes during development was observed on the head (Fig. 4C) and trunk (Fig. 4E), but not on the yolk sac (Fig. 4D). This was the case for 5-HT2A-positive ionocytes co-labelled with Mitotracker (Fig. 4H,K) as well as ionocytes that were 5-HT2A-positive but Mitotracker negative (Fig. 4G,J) on the head and trunk. Cells that were positive for Mitotracker only were very few in number and never demonstrated a significant change with acid exposure (Fig. 4F,I). Given that they did not respond to treatment and did not contain 5-HT2A, these cells were not quantified in the remaining experiments. Compared with other regions, ionocytes on the trunk were best labelled and most evenly distributed, so only the trunk was used to quantify the change in ionocyte number in subsequent experiments. In addition, fish demonstrated a robust increase in ionocyte number after 6 days of exposure to acidic media (at 6 dpf); therefore, this time point was used in subsequent experiments.

### The number of 5-HT2A-positive ionocytes increases with exposure to 5-HT, but not with the addition of ketanserin

**Fig. 5.**
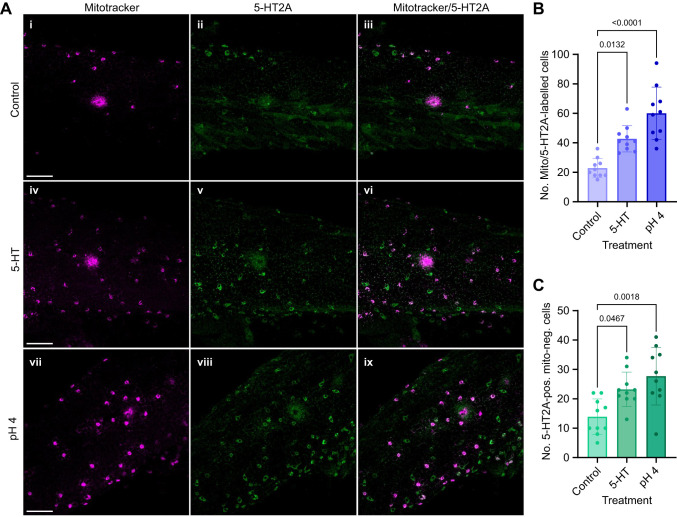
**5-HT exposure resulted in an increased number of 5-HT2A-positive ionocytes.** (A) Representative confocal images of Mitotracker (magenta) labelling (i,iv,vii), 5-HT2A (green) labelling (ii,v,viii) and Mitotracker-5/HT2A (white) co-labelling (iii,vi,ix) on the trunk of zebrafish larvae at 6 dpf in control medium (i–iii), medium supplemented with 100 μmol l^−1^ 5-HT (iv–vi) and at pH 4 (vii–ix). Fish were treated for 6 days (from 0 to 6 dpf). Scale bars in A (across rows): 50 μm. (B) The number of ionocytes co-labelled with Mitotracker and 5-HT2A. (C) The number of ionocytes labelled with 5-HT2A, but not with Mitotracker. Data were analysed using a Kruskal–Wallis test (two-tailed) with Dunn's multiple comparison test. *P*-values are shown on the graph for groups that are significantly different from the control (*P*<0.05, *N*=10 for each group). Data are means±s.d.

Following the identification of 5-HT2A in proliferating ionocytes, we sought to investigate a role for the 5-HT2A receptor in increasing ionocyte number. Considering this hypothesis, we first exposed zebrafish larvae to the agonist, 5-HT, for 6 days (from 0 to 6 dpf). Following 5-HT exposure, the number of 5-HT2A-positive ionocytes was significantly increased (*P*<0.05, *N*=10), regardless of whether they were co-labelled with Mitotracker, as was the case for ionocyte number in pH 4-treated fish (Fig. 5B,C).

**Fig. 6.**
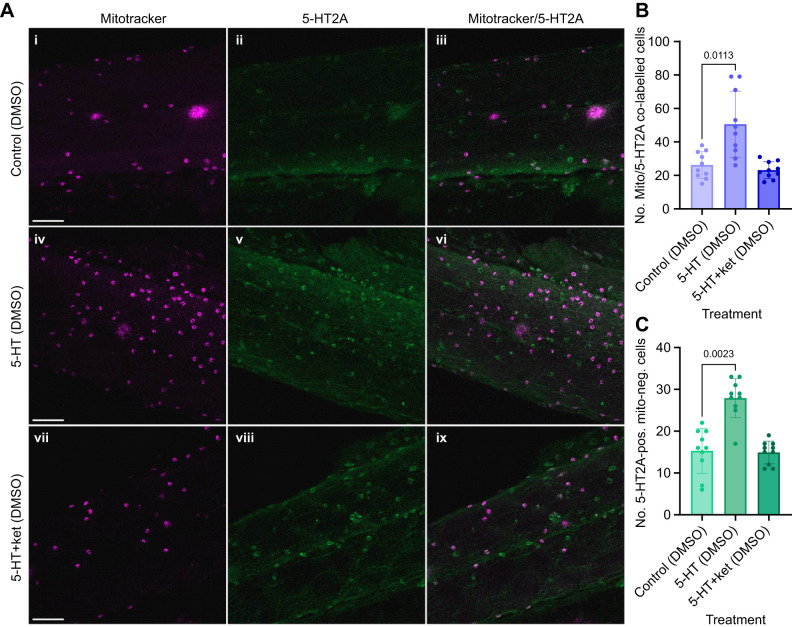
**Pre-treatment with ketanserin inhibited the increase in ionocyte number elicited by 5-HT.** (A) Representative confocal images of Mitotracker (magenta) labelling (i,iv,vii), 5-HT2A (green) labelling (ii,v,viii), and Mitotracker/5-HT2A (white) co-labelling (iii,vi,ix) on the trunk of zebrafish larvae at 6 dpf in control medium supplemented with 0.1% DMSO (i–iii), in medium supplemented with 100 μmol l^−1^ 5-HT and 0.1% DMSO (iv–vi) and in medium with 100 μmol l^−1^ 5-HT following pre-treatment with 100 μmol l^−1^ ketanserin supplemented with 0.1% DMSO (vii–ix). Fish were treated for 6 days (from 0 to 6 dpf). Scale bars in A (across rows): 50 μm. (B) The number of ionocytes co-labelled with Mitotracker and 5-HT2A. (C) The number of ionocytes labelled with 5-HT2A, but not Mitotracker. Data were analysed using a Kruskal–Wallis test (two-tailed) with Dunn's multiple comparison test. *P*-values are shown on the graph for groups that are significantly different from the control (*P*>0.05, *N*=10 for each group). Data are means±s.d.

In a subsequent round of experiments, we demonstrated that 5-HT was acting specifically through the 5-HT2A receptor to cause the increase in the number of ionocytes. We pre-exposed zebrafish larvae to the specific 5-HT2A antagonist ketanserin, before exposing them to 5-HT. When fish were pre-exposed to ketanserin, the effect of 5-HT on ionocyte number was abolished, while 5-HT treatment in the absence of ketanserin continued to have a significant effect (*P*<0.05, *N*=10) (Fig. 6B,C). These experiments included solutions supplemented with 0.1% DMSO, as it was required to dissolve ketanserin. DMSO itself had no effect on ionocyte number (Fig. S2).

### Treatment with 5-HT results in phosphorylation of ERK in ionocytes, which is reduced when fish are pre-treated with ketanserin

**Fig. 7.**
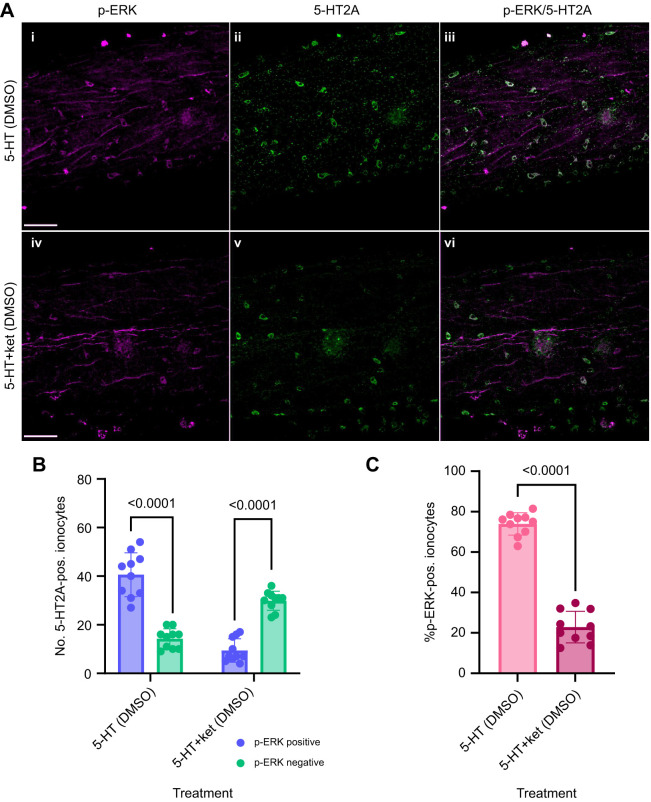
**A higher percentage of ionocytes contained phosphorylated extracellular signal-regulated kinase (p-ERK) when larvae were treated with 5-HT than when they were pre-treated with ketanserin.** (A) Representative confocal images of p-ERK (magenta) labelling (i,iv), 5-HT2A (green) labelling (ii,v) and p-ERK/5-HT2A (white) co-labelling (iii,vi) on the trunk of zebrafish larvae at 6 dpf in 100 μmol l^−1^ 5-HT supplemented with 0.1% DMSO (i–iii) and in 100 μmol l^−1^ 5-HT following pre-treatment with 100 μmol l^−1^ ketanserin supplemented with 0.1% DMSO (iv–vi). Fish were treated for 6 days (from 0 to 6 dpf). Scale bars in A (across rows): 50 μm. (B) The number of p-ERK-positive ionocytes compared with the number of p-ERK-negative ionocytes in each treatment group. (C) The percentage of p-ERK-labelled 5-HT2A-positive ionocytes in each treatment group. Data in B were analysed using a Multiple Mann–Whitney test (two-tailed) with the two-stage linear set-up procedure of Benjamini, Krieger and Yekutieli. Data in C were analysed using a Mann–Whitney test (two-tailed). *P*-values are shown on the graph for groups that are significantly different (*P*<0.05, *N*=10 for each group). Data are means±s.d.

Given that ERK is commonly involved in signalling cascades initiated by the 5-HT2A receptor, we hypothesized that treatment with 5-HT would result in phosphorylation of ERK in 5-HT2A-positive ionocytes. We used an antibody raised against p-ERK to demonstrate activation of a pathway in ionocytes upon stimulation of 5-HT2A with 5-HT. Fish were processed for immunohistochemistry using anti-p-ERK and anti-5-HT2A antibodies. We quantified the number of p-ERK-positive and negative ionocytes in each fish and, subsequently, the percentage of 5-HT2A-positive ionocytes co-labelled with p-ERK (Fig. 7). In unstimulated controls, no clear p-ERK labelling was observed in any ionocytes. However, in 5-HT and ketanserin-treated samples, p-ERK-positive cells were observed (Fig. 7). In 5-HT-treated samples, the number of p-ERK-positive ionocytes was significantly greater (*P*<0.0001, *N*=10) than the number of p-ERK-negative ionocytes (Fig. 7B). In fish pre-treated with ketanserin, the number of p-ERK-positive ionocytes was significantly lower (*P*<0.0001, *N*=10) than the number of p-ERK negative ionocytes (Fig. 7B). Furthermore, in 5-HT-treated samples, an average of about 74% of 5-HT2A-labelled ionocytes were p-ERK positive, which was significantly reduced (*P*<0.0001, *N*=10) to an average of about 23%, when fish were pretreated with ketanserin (Fig. 7C).

### Ketanserin and tetrabenazine inhibit the observed increase in ionocyte number in acidic medium

**Fig. 8.**
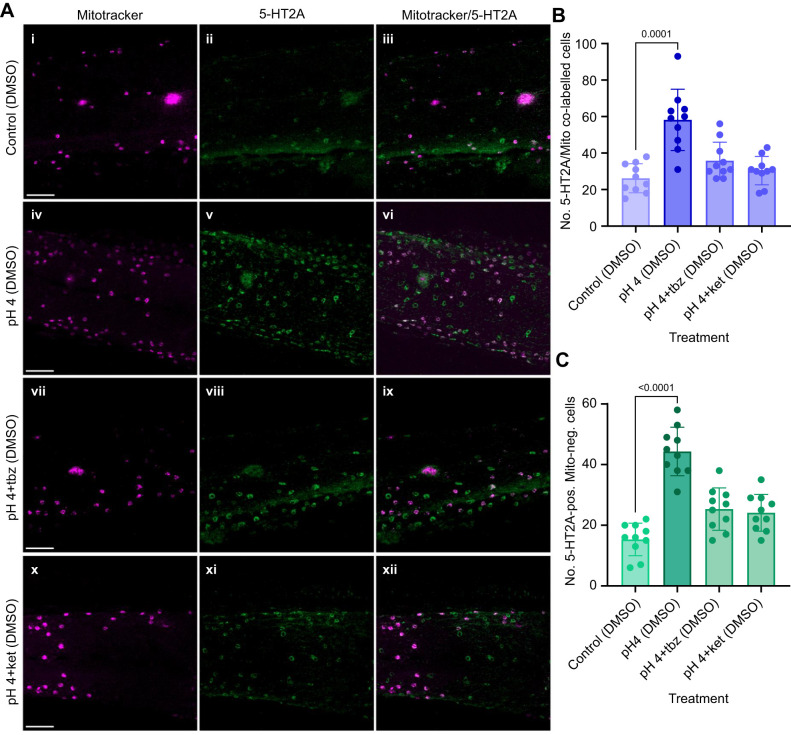
**Treatment with ketanserin or tetrabenazine inhibited the increase in ionocyte number in acid-treated fish.** (A) Representative confocal images of Mitotracker (magenta) labelling (i,iv,vii,x), 5-HT2A (green) labelling (ii,v,viii,xi) and Mitotracker/5-HT2A (white) co-labelling (iii,vi,ix,xii) on the trunk of zebrafish larvae at 6 dpf in control medium with 0.1% DMSO (i–iii), pH 4 medium with 0.1% DMSO (iv–vi), pH 4 medium supplemented with 100 μmol l^−1^ tetrabenazine and 0.1% DMSO (vii–ix), and pH 4 medium supplemented with 100 μmol l^−1^ ketanserin and 0.1% DMSO (x–xii), conditions. Fish were treated for 6 days (from 0 to 6 dpf). Scale bars in A (across rows): 50 μm. (B) The number of ionocytes co-labelled with Mitotracker and 5-HT2A. (C) The number of ionocytes labelled with 5-HT2A only. Data were analysed using a Kruskal–Wallis test (two-tailed) with Dunn's multiple comparison test. *P*-values are shown on the graph for groups that are significantly different from the control (*P*<0.05, *N*=10 for each group). Controls for B and C were performed at the same time as those for Fig. 7 and are derived from the same dataset. Data are means±s.d.

Given that ketanserin inhibited the increase in ionocyte number observed in the presence of 5-HT, we next sought to investigate whether it could also inhibit the increase in ionocyte number with acclimation to an acidic environment (Fig. 8). Following exposure to acidic medium supplemented with ketanserin from 0 to 6 dpf, no significant increase (*P*>0.05, *N*=10) in ionocyte number was observed compared with the negative control (Fig. 8B,C).

Having demonstrated that 5-HT2A receptors are involved in the proliferation of ionocytes in an acidic environment, we also sought to provide evidence for an endogenous source of 5-HT, such as from nearby cutaneous NECs, that may be activating this process. We used tetrabenazine to prevent the release of 5-HT from potential nearby sources. Tetrabenazine disrupts the loading and storage of monoamines through inhibition of vmat2 and has been shown to inhibit 5-HT-mediated signalling in gill NECs in zebrafish (Reed et al., 2025). When exposed to acidic medium supplemented with tetrabenazine, larvae demonstrated no significant increase (*P*>0.05, *N*=10) in ionocyte number compared with the negative control (Fig. 8B,C).

The number of ionocytes remained significantly upregulated in acid-treated fish in the absence of ketanserin and tetrabenazine for cells co-labelled with 5-HT2A and Mitotracker, and cells positive for 5-HT2A only (*P*=0.0001 and *P*<0.0001 respectively, *N*=10) (Fig. 8B,C).

**Fig. 9.**
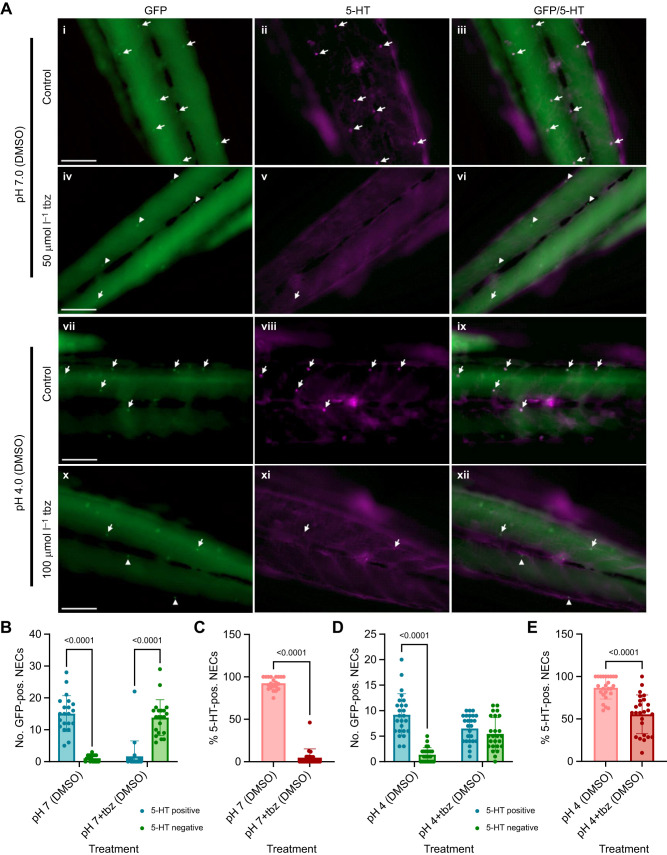
**Tetrabenazine treatment depleted cutaneous neuroepithelial cells of 5-HT at pH 7.0 and at pH 4.0.** (A) Representative epifluorescence images of GFP (green) labelling (i,iv,vii,x), 5-HT (magenta) labelling (ii,v,viii,xi) and GFP/5-HT (white) co-labelling (iii,vi,ix,xii) on the trunk of zebrafish larvae at 4 dpf in pH 7.0 control media with 0.1% DMSO (i–iii), pH 4.0 control media with 0.1% DMSO (vii–ix), pH 7.0 media supplemented with 50 μmol l^−1^ tetrabenazine and 0.1% DMSO (iv–vi), or pH 4.0 media supplemented with 100 μmol l^−1^ tetrabenazine and 0.1% DMSO (x–xii) conditions. Fish were treated for 4 days (from 0 to 4 dpf). Scale bars in A (across rows): 100 μm. Arrows point to neuroepithelial cells (NECs) that demonstrate 5-HT labelling, arrowheads point to NECs that are 5-HT negative. (B,D) The number of 5-HT-positive compared with 5-HT-negative NECs in control versus treatment groups at pH 7.0 (B) and pH 4.0 (D). (C,E) The percentage of 5-HT-positive NECs in each treatment group at pH 7.0 (C) and pH 4.0 (E). Data in B and D were analysed using a Multiple Mann–Whitney test (two-tailed) with the two-stage linear set-up procedure of Benjamini, Krieger and Yekutieli. Data in C and E were analysed using a Mann–Whitney test (two-tailed). *P*-values are shown on the graph for groups that are significantly different (*P*<0.05, *N*=21 for pH 7.0 and *N*=25 for pH 4.0). Data are means±s.d.

To confirm that tetrabenazine can deplete cutaneous NECs in zebrafish larvae of 5-HT, and that it maintains its effect at pH 4.0, we immunohistochemically labelled 5-HT in ET(vmat2:GFP) zebrafish larvae at 4 dpf (Fig. 9). There were significantly more 5-HT-positive NECs than 5-HT-negative NECs in control groups at both pH 7.0 and pH 4.0 (*P*<0.0001, *N*=21 at pH 7.0 and *P*<0.0001, *N*=25 at pH 4.0), while following tetrabenazine treatment there were significantly fewer 5-HT-positive NECs than 5-HT-negative NECs at pH 7.0 (*P*<0.0001, *N*=21) (Fig. 9B) and no significant difference between the number of 5-HT-positive and 5-HT-negative NECs at pH 4.0 (*P*>0.05, *N*=25) (Fig. 9D). Additionally, we observed a significant decrease in the percentage of GFP-labelled (i.e. vmat2-containing) NECs that stained positive for 5-HT following treatment with tetrabenazine at pH 7.0 (*P*<0.0001, *N*=21) (Fig. 9C), which remained the case at pH 4.0 (*P*<0.0001, *N*=25) (Fig. 9E).

## DISCUSSION

**Fig. 10.**
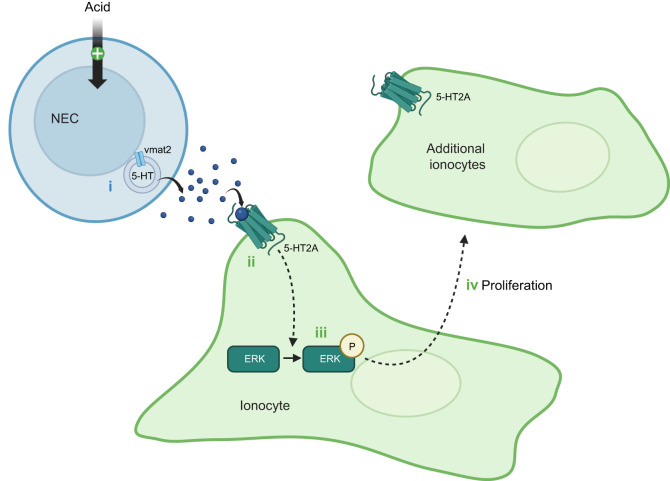
**Model of a proposed pathway through which acid acclimation may lead to cutaneous ionocyte proliferation in zebrafish.** (i) Serotonin (5-HT) is loaded into secretory vesicles (in this case in a neuroepithelial cell, NEC) by the vesicular monoamine transporter 2 (vmat2) and is stored until released by acid stimulation. The only known source of cutaneous 5-HT in developing zebrafish is chemosensitive NECs (Coccimiglio and Jonz, 2012). (ii) 5-HT binds to and activates 5-HT2A receptors on nearby ionocytes to initiate an intracellular signalling cascade. (iii) The signalling cascade results in phosphorylation (P) of extracellular signal-regulated kinase (ERK), a regulator of cell division and differentiation. (iv) Phosphorylated ERK leads to the proliferation of new 5-HT2A-positive ionocytes. See Discussion for further details. Created with BioRender by Jonz, M., 2026. https://BioRender.com/066xsse. This figure was sublicensed under CC-BY 4.0 terms.

This study presents the novel finding that cutaneous ionocytes in developing zebrafish express 5-HT2A receptors. We have provided evidence that the increase in ionocyte number following acid acclimation is mediated, at least in part, by activation of 5-HT2A. A proposed scheme for a pathway through which 5-HT mediates ionocyte proliferation is discussed in the following sections and is summarized in Fig. 10.

### Ionocytes contain 5-HT2A receptors

The immunohistochemical data from experiments using α5, ConA and Mitotracker demonstrate constitutive expression of 5-HT2A receptors in NaR and HR cells, as well as in MRCs. Furthermore, given the near-complete co-localization of 5-HT2A labelling with Mitotracker dye, it seems likely that all cutaneous ionocyte subtypes contain 5-HT2A in zebrafish larvae until at least 7 dpf. Some cells reported in the present study were positive for 5-HT2A but did not take up Mitotracker. Despite not being co-labelled with this common ionocyte marker, these cells were ionocyte-like based on their morphology, distribution and response to acidic stimuli. This population represented approximately 24% of 5-HT2A-positive ionocytes and may correspond to SLC26 and KS ionocytes, which have not yet been demonstrated to stain with Mitotracker. Alternatively, these cells may be MRCs. It is important to note that Mitotracker dyes have been shown to be inconsistent in some cases (Neikirk et al., 2023) and may underestimate the labelling of cells that are rich in mitochondria, particularly in whole-mount preparations, as in the present study. We continued to differentiate between 5-HT2A-positive ionocytes, with and without Mitotracker labelling, and in each case all 5-HT2A-positive, ionocyte-like cells responded the same way to stimuli. This robust increase in cell number following environmental acidification suggests that the 5-HT2A-positive Mitotracker-negative population of cells are involved in a physiological response to acid exposure. Future studies on the expression profile of these cells and their role in the maintenance of osmotic homeostasis would be required to definitively identify this group as a population of ionocytes.

### Activation of 5-HT2A receptors mediates an increase in ionocyte number

The effects of 5-HT on ionocyte activity have not previously been recognized. In one study, Kumai et al. (2012) demonstrated that 5-HT had no effect on Na^+^ uptake in HR cells in zebrafish. We hypothesized that 5-HT2A receptors play a role in ionocyte proliferation. The increase in ionocyte number following exposure to 5-HT, and inhibition of this effect by pre-exposure to ketanserin, a 5-HT2A receptor antagonist, strongly suggest that this is the case.

This finding agrees with the literature demonstrating that 5-HT receptors are regulators of the cell cycle. A review by Azmitia (2001) highlights multiple examples of 5-HT2A and 5-HT1A receptors in the regulation of cell proliferation, maturation and apoptosis. Similarly, Masson et al. (2012) discussed a mitogenic role for 5-HT2A receptors, highlighting induction of growth of smooth muscle cells and potentiation of the activity of growth factors. Furthermore, 5-HT, via 5-HT2A receptors, has known roles in facilitating the proliferation of neurons in the developing neocortex (Xing et al., 2020), is known for its mitogenic effects on trophoblast cells (Fecteau and Eiler, 2001; Oufkir et al., 2010; Sonier et al., 2005), and has a proliferative effect on rat renal mesangial cells (Takuwa et al., 1989).

The signalling pathways through which 5-HT2A receptors lead to cell proliferation are complex, but many examples implicate phosphorylation of ERK (Göőz et al., 2006; Greene et al., 2000; Grewal et al., 1999; Masson et al., 2012; Oufkir et al., 2010). We found that approximately 74% of 5-HT2A-positive ionocytes contained p-ERK in 5-HT-treated fish, and this effect was reduced to about 23% when fish were pre-treated with ketanserin. These results implicate phosphorylation of ERK as part of a signalling pathway through which 5-HT2A receptor activation increases ionocyte number in zebrafish larvae. Furthermore, the presence of p-ERK in ionocytes stimulated with 5-HT provides direct evidence for the activation of a pathway in 5-HT2A-positive ionocytes.

### 5-HT2A receptor activation occurs in response to environmental acidification

Given that 5-HT levels are known to increase in the brain (Boeck et al., 1996; Sreelekshmi et al., 2022) and gills (Mustafayev and Mekhtiev, 2008) of fishes exposed to osmotic stress, one of the aims of our study was to uncover whether 5-HT mediates the increase in ionocyte number under similar conditions. This hypothesis was supported by our demonstration that ionocyte proliferation, in response to an acidic environment, was inhibited by tetrabenazine and ketanserin, which deplete 5-HT stores and inhibit 5-HT2A, respectively. These findings demonstrate for the first time that 5-HT, via the 5-HT2A receptor, plays a role in ionocyte proliferation as zebrafish larvae acclimate to an acidic environment. These findings may extend further to ionoregulatory mechanisms which are tightly linked to acid–base homeostasis. Interestingly, in juvenile carp, increasing dietary tryptophan (a precursor of 5-HT) has previously been demonstrated to result in increased salinity tolerance (Hoseini and Hosseini, 2010). It is not yet known whether ionocytes in carp contain 5-HT2A receptors, but future studies may determine whether ionocyte proliferation due to increased 5-HT production may have implications for the observed increase in tolerance to osmotic stress.

Our results suggest that release of 5-HT mediates the increase in ionocyte number in response to acid exposure. The source of 5-HT, however, remains to be definitively elucidated. Chemosensitive NECs are promising candidates for the regulation of cutaneous ionocyte proliferation because they are the only known source of cutaneous 5-HT in developing zebrafish. NECs store 5-HT, express vmat2 and are found in close proximity to ionocytes in the skin and gills in zebrafish (Coccimiglio and Jonz, 2012; Dean et al., 2017; Jonz and Nurse, 2006; Pan et al., 2021). In the gills, 5-HT depletion by tetrabenazine inhibits serotonergic signalling from NECs to postsynaptic neurons that express 5-HT3 receptors (Reed et al., 2025). Moreover, gill NECs express acid-sensitive TASK-2 ion channels (Peña-Münzenmayer et al., 2014) and have been shown to produce excitatory signals in response to extracellular H^+^ (Abdallah et al., 2015), a response that leads to the neurosecretion of 5-HT from NECs (Reed et al., 2025). We demonstrate here that 50 μmol l^−1^ tetrabenazine is sufficient to nearly abolish 5-HT labelling of cutaneous NECs at pH 7.0 with only about 5% of NECs demonstrating 5-HT labelling, while at pH 4.0, 100 μmol l^−1^ tetrabenazine reduced the percentage of 5-HT-positive NECs to about 50%. This demonstrates that the effect of tetrabenazine is reduced at pH 4.0, but is not abolished at the 100 μmol l^−1^ concentration used. Furthermore, while we deemed NECs either 5-HT-positive or 5-HT-negative, we expect based on the reduced fluorescence intensity of 5-HT labelling in tebtrabenazine-treated fish that the 5-HT-positive NECs may have been partially depleted. Our attribution of 50% depletion of NECs at pH 4.0 is therefore likely an underestimate of the effect of the drug. Interestingly, 5-HT labelling also appeared less intense, and there appeared to be more 5-HT-negative NECs in control fish at pH 4.0 compared with control fish at pH 7.0, which may be a result of the proposed 5-HT release from NECs upon acid exposure.

In the present study, we confirm that 100 μmol l^−1^ tetrabenazine depletes cutaneous NECs of 5-HT when exogenously applied at pH 4.0, and inhibits ionocyte proliferation elicited by environmental acidification. Combined, these results suggest that 5-HT storage and release from NECs may be an integral part of regulating ionocyte proliferation. Direct evidence demonstrating 5-HT release from cutaneous NECs in response to acid exposure remains an important question for future investigation.

### Implications and conclusions

As we continue to gain insight into the complex mechanisms that underly ionocyte proliferation in response to environmental acidification, definitive identification of the cell type that releases 5-HT to activate this pathway will be paramount, as will the identification of the specific source of increased ionocyte number. It has been suggested by Horng et al. (2009) that additional HR cells in pH 4-exposed fish may differentiate from proliferating p63-positive epithelial stem cells or other ionocyte precursors. Should this be the case for all ionocytes, the signalling cascade downstream of p-ERK may involve spatial propagation of ERK signals from activated ionocytes to ionocyte precursors, leading to the regulation of proliferation and differentiation of ionocyte precursors to elicit the increase in ionocyte number. There is precedence for such a mechanism in the mammalian epidermis, with implications in the regulation of cell density during development and wound healing (Aoki et al., 2013; Hiratsuka et al., 2015, 2020). Alternatively, it is possible that the signalling cascade initiated by 5-HT2A results in the direct proliferation of ionocytes. To differentiate between these possibilities, more studies on the cellular origins of ionocytes in acid-acclimated zebrafish larvae, and expression profiles of ionocyte precursors, would be required. Such experiments may also shed light on which specific ionocyte subtypes are proliferating in response to acid exposure.

Furthermore, identification of the remaining molecular players upstream and downstream of p-ERK will allow further comparisons to be drawn between ionocytes and other cell types in vertebrates. As our understanding of this pathway expands, this research may have implications for the use of zebrafish as a model organism, should the pathway continue to present parallels with similar cell types in mammals. Finally, investigation of the presence of 5-HT2A and the importance of this pathway in gill ionocytes of adult zebrafish and in ionocytes of other fish species is warranted. Should the same mechanism be present in ionocytes in other fish species, this research may have applications for fish rearing in osmotically stressful environments.

## Supplementary Material

10.1242/jexbio.251808_sup1 Supplementary information
