# Supplementary material for: Serotonin stimulates proliferation of ionocytes via the 5-HT2A receptor in zebrafish larvae
Source: J Exp Biol. 2026 Jun 15;229(12):jeb251808. doi: 10.1242/jeb.251808 (PMC13327541; doi:10.1242/jeb.251808)
Supplement: Supplementary information [file jexbio-229-251808-s1.pdf]

```
5-HT2Ab         MALNGT--A----AELRSLGISAEISKDTSMEQKVSLGNAVLNNSMDCNGS--------- 45
5-HT2AaX2       ------------------------------------------------------------ 0
5-HT2AaX1       MNLHVNISKPVPKSVLTSSNLMPFDPDSWTPSPDIMLGNVVHNSSIGCNRSWTDTEASLL 60
Antigen         ------------------------------------------------------------ 0

5-HT2Ab         -----------------EERNRSEFSSSDYVQKNWVALLISLVIIITVTGNILVIMAVSL 88
5-HT2AaX2       ------------------------------------------------------------ 0
5-HT2AaX1       PNLSMIGISSERTELLSSQRCNGETTKEELVRKNWAALLILVVVIVTVAGNILVIMAVNL 120
Antigen         ------------------------------------------------------------ 0

5-HT2Ab         ERKLQNATNYFLRSLAITDMLLGILVMPVAMVTILYGYTWPLPRTLCPIWIYLDVLFSTA 148
5-HT2AaX2       ------------------------------------------------MWIYLDVLFSTA 12
5-HT2AaX1       ERKLQNATNYFLMSLAVADMLLGLLVMPVSMVTIVYGYSWPFPASLCPMWIYLDVLFSTA 180
Antigen         ------------------------------------------------------------ 0

5-HT2Ab         SIMHLCAISLDRYIAIRNPIHHSRSNSLTKARVKIIAAWTISVVISMPVPVLGLHDHSKV 208
5-HT2AaX2       SIMHLCAISLDRYVAIRNPIRHNRSNSRSRARAKITAVWTISAGISMPIPVLGLRDHTKV 72
5-HT2AaX1       SIMHLCAISLDRYVAIRNPIRHNRSNSRSRARAKITAVWTISAGISMPIPVLGLRDHTKV 240
Antigen         ------------------------------------------------------------ 0

5-HT2Ab         FRNESCQLTDNNFVLIGSFVAFFVPLIIMVVTYFLTISALQSEATLCLDQLIVRPTWSST 268
5-HT2AaX2       FKDGSCLLTDNSFVLIGSFVAFFVPLTIMVVTYFLTISALQSEATLCLDQLVPRPKWSTG 132
5-HT2AaX1       FKDGSCLLTDNSFVLIGSFVAFFVPLTIMVVTYFLTISALQSEATLCLDQLVPRPKWSTG 300
Antigen         ------------------------------------------------------------ 0

5-HT2Ab         IGL--LPRG--SVSSERLFSRSSICREGA------SRGSGRRSMQSISNEQKASKVLGVV 318
5-HT2AaX2       LTLNFLPGPSFSPSEKKLFLRRSLSREPGADSGVVTPPFGRHNMQSISNEQKASKVLGVV 192
5-HT2AaX1       LTLNFLPGPSFSPSEKKLFLRRSLSREPGADSGVVTPPFGRHNMQSISNEQKASKVLGVV 360
Antigen         ------------------------------------------------------------ 0

5-HT2Ab         FLLFVIMWCPFFVTNVMAVVCGS-VCDEDLVGGLMNVFVWVGYLSSAVNPFIYTLFNKTY 377
5-HT2AaX2       FFLFVVMWCPFFITNVLAVVCEPNACNANIMNRLLNVFVWVGYLSSAVNPLVYTLFNKTY 252
5-HT2AaX1       FFLFVVMWCPFFITNVLAVVCEPNACNANIMNRLLNVFVWVGYLSSAVNPLVYTLFNKTY 420
Antigen         ------------------------------------------------------------ 0

5-HT2Ab         RAAFARYMQCRYHEERRPLQLILVNTIPPLAYSSSGLPLKVENSLRRKAEGSRSGSFTNT 437
5-HT2AaX2       RSAFARYIRCQFHEEKKPLQLILVNTIPPMAYQSTHLPLTGSIGN-----GDFSLPLPNK 307
5-HT2AaX1       RSAFARYIRCQFHEEKKPLQLILVNTIPPMAYQSTHLPLTGSIGN-----GDFSLPLPNK 475
Antigen         ------------KENKKPLQLILVNTIPALAYKSSQLQMGQKKN---------------- 32
                            :*:::*********** :**.*: * :  . .

5-HT2Ab         ERSICGSTQNKQERDEVVSHL  458
5-HT2AaX2       NHHLS-----KSGKNESVSCL  323
5-HT2AaX1       NHHLS-----KSGKNESVSCL  491
Antigen         ---------------------  32
```

Fig. S1. Sequence alignment for the antigen of the 5-HT2A antibody compared to zebrafish 5-HT2A receptor paralogs and isoforms. Sequence alignment from ClustalOmega (URL: https://www.ebi.ac.uk/jdispatcher/msa/clustalo, accessed Jan. 5, 2026) for the antigen of the 5-HT2A antibody (manufacturer specifications) with the sequence of the zebrafish (*Danio rerio*) 5-HT2A receptor genome duplicate a, isoform X1 (5-HT2AaX1) (NCBI reference sequence XP_689300.7), genome duplicate a, isoform X2 (5-HT2AaX2) (NCBI reference sequence XP_068079731.1) and genome duplicate b (5-HT2Ab) (NCBI reference sequence XP_073809815.1) obtained from NCBI (URL: https://www.ncbi.nlm.nih.gov/, accessed Jan. 5, 2025). (.) represents semi-conservative substitutions, (:) conserved substitutions and (*) fully conserved residues.

Journal of Experimental Biology • Supplementary information

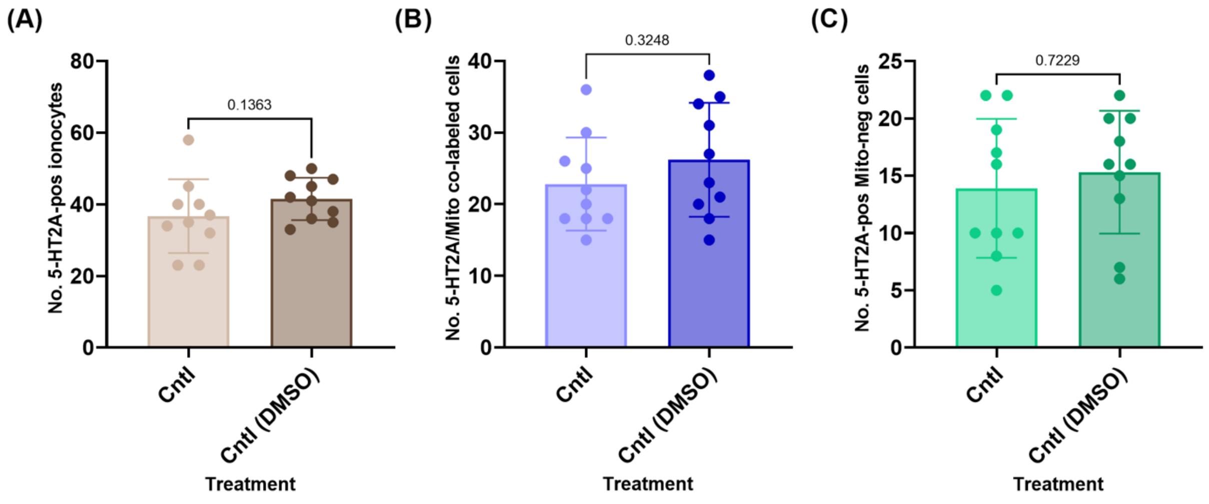

Fig. S2. DMSO did not significantly change the number of 5-HT2A-positive ionocytes. The addition of 0.1% DMSO did not significantly alter ($p>0.05$, N=10) (A) the total number of 5-HT2A-positive ionocytes, (B) the number of ionocytes co-labeled with 5-HT2A and Mitotracker, or (C) the number of 5-HT2A-positive ionocytes that were Mitotracker-negative. Fish treated without DMSO (Control, Cntl) and with DMSO were exposed and processed for immunohistochemistry. Data analyzed using a Mann-Whitney test (two-tailed) ($p<0.05$, N=10 for each group). All p values are shown on the graphs. Average values represented as mean ± s.d. 5-HT, serotonin; 5-HT2A, serotonin 2A receptor; DMSO, dimethyl sulfoxide; pos, positive.

Journal of Experimental Biology • Supplementary information
